# Impact of localized fine tuning in the performance of segmentation and classification of lung nodules from computed tomography scans using deep learning

**DOI:** 10.3389/fonc.2023.1140635

**Published:** 2023-03-28

**Authors:** Jingwei Cai, Lin Guo, Litong Zhu, Li Xia, Lingjun Qian, Yuan-Ming Fleming Lure, Xiaoping Yin

**Affiliations:** ^1^ Radiology Department, Affiliated Hospital of Hebei University, Baoding, Hebei, China; ^2^ Clinical Medical College, Hebei University, Baoding, Hebei, China; ^3^ Shenzhen Zhiying Medical Imaging, Shenzhen, Guangdong, China; ^4^ Department of Medicine, Queen Mary Hospital, University of Hong, Hong Kong, Hong Kong SAR, China

**Keywords:** segmentation, classification, lung nodules, localized fine tuning, site-specific use

## Abstract

**Background:**

Algorithm malfunction may occur when there is a performance mismatch between the dataset with which it was developed and the dataset on which it was deployed.

**Methods:**

A baseline segmentation algorithm and a baseline classification algorithm were developed using public dataset of Lung Image Database Consortium to detect benign and malignant nodules, and two additional external datasets (i.e., HB and XZ) including 542 cases and 486 cases were involved for the independent validation of these two algorithms. To explore the impact of localized fine tuning on the individual segmentation and classification process, the baseline algorithms were fine tuned with CT scans of HB and XZ datasets, respectively, and the performance of the fine tuned algorithms was tested to compare with the baseline algorithms.

**Results:**

The proposed baseline algorithms of both segmentation and classification experienced a drop when directly deployed in external HB and XZ datasets. Comparing with the baseline validation results in nodule segmentation, the fine tuned segmentation algorithm obtained better performance in Dice coefficient, Intersection over Union, and Average Surface Distance in HB dataset (0.593 vs. 0.444; 0.450 vs. 0.348; 0.283 vs. 0.304) and XZ dataset (0.601 vs. 0.486; 0.482 vs. 0.378; 0.225 vs. 0.358). Similarly, comparing with the baseline validation results in benign and malignant nodule classification, the fine tuned classification algorithm had improved area under the receiver operating characteristic curve value, accuracy, and F1 score in HB dataset (0.851 vs. 0.812; 0.813 vs. 0.769; 0.852 vs. 0.822) and XZ dataset (0.724 vs. 0.668; 0.696 vs. 0.617; 0.737 vs. 0.668).

**Conclusions:**

The external validation performance of localized fine tuned algorithms outperformed the baseline algorithms in both segmentation process and classification process, which showed that localized fine tuning may be an effective way to enable a baseline algorithm generalize to site-specific use.

## 1 Introduction

Lung cancer is one of the most common cancers in the world (1), which has no obvious clinical symptoms in the early stage, but is hardly cured after the onset of disease. Therefore, early diagnosis and differentiation of benign and malignant pulmonary nodules has great significance for the long-term survival of patients (2). As one of the most important means to screen lung cancer for high-risk groups (3), low-dose CT scans have been widely used in health examinations, and a large amount of CT data has created heavy workload for radiologists. Deep learning (DL) is considered as a powerful tool that have gained great achievements in the detection of benign and malignant pulmonary nodules in chest CT images (4, 5). However, in most cases, decreased performance is observed when the proposed algorithm is applied in the external tests, even with adopted and balanced validation datasets (6–9).

It has been a public concern that algorithm malfunction occurs when it is applied on external dataset that is inherently different from the training set. It may halt the possible implementation of the general model into routine clinical care if it does not have a consistent accuracy for site-specific use. To obtain a comparable external test performance to the internal tests, reported studies involving training datasets from multicenter to develop the detection algorithm demonstrated that it can either underperform (10–12) or have a comparable performance to the internal test (11, 13) without any unanimous conclusion reached, which may be explained by the differences of the datasets scale and the numbers of dataset origins (14). Using local images for model training seems to be another way to obtain a site-specific used tool for diagnosis. However, a large amount of training images is needed to develop a DL algorithm, which is challenging for those regions with lower prevalence of lung nodules, especially malignant nodules. Therefore, developing a baseline algorithm using only public dataset and then recalibrating it with local images may be an effective way to reduce site-specific bias.

It has been proved that recalibration strategy with local data is able to correct for the anticipated drop in model performance. Various studies related to recalibration method were reported, but in most cases, they are statistical prediction models focusing on updating regression coefficients, or adding new covariates for the model (15–18). To the best of our knowledge, few studies have been conducted with recalibration strategy of localized fine tuning on imaging to separately explore its impact on the segmentation and classification process.

In the study, we conducted localized fine tuning for the baseline DL algorithm of segmentation and classification to segment and classify benign and malignant nodules. The baseline algorithms were first developed using public dataset of Lung Image Database Consortium (LIDC) (19) and then 50% of the public data was replaced with local dataset to develop the fine tuned algorithms. The performance of the fine tuned algorithms and baseline algorithms were tested and compared in multicenter datasets.

## 2 Methods

### 2.1 Patient cohorts

The studies involving human participants were reviewed and approved by the Institutional Review Board (IRB) of the Affiliated Hospital of Hebei University. The informed consent from human participants was waived because this is a retrospective study, and the waiver was indicated in the IRB approval document. Three datasets were involved in the study, including a public dataset of LIDC and two collected datasets named HB and XZ, respectively. All identifications of the patient were removed.

LIDC has a total of 1018 cases (the number of patients was unknown) with annotation process performed by four radiologists. Each radiologist independently reviewed the CT images and marked lesions that belonged to one of three categories (“nodule >or = 3 mm”, “nodule < 3 mm” and “non-nodule >or = 3 mm”). The nodules are finally marked with 5 malignancy levels, from 1 to 5 (17). As the detection algorithm was developed for the nodule-level classification, the inclusion criteria for nodules are as follows: (1) Nodule diameter >3mm; (2) Nodules with score greater than 3 were included with malignant label, and nodules with score less than 3 were included with benign label; (3) Nodules with borderline median malignancy (rating =3) were excluded; (4) Nodules with only one score were excluded. Finally, 582 cases comprising of 430 malignant nodules and 671 benign nodules were included, and they were randomly divided into training and testing set at a ratio of 8:2; the training set contained 344 malignant nodules and 536 benign nodules, and the testing set contained 86 malignant nodules and 135 benign nodules.

A total of 541 patients in HB dataset were retrospectively collected from January 2017 to June 2020, and 261 patients in XZ dataset were collected from July 2019 to May 2020. The inclusion criteria for these two datasets were: (1) The patients had typical imaging signs and pathological results of the lesions; (2) There was no surgery in the lung; (3) There was no history of malignant tumor in other part of the lung. Finally, a total of 963 nodules of HB dataset were included, comprising of 537 malignant nodules and 426 benign nodules, and a total of 785 nodules in XZ dataset with 387 malignant nodules and 398 benign nodules were also involved.

### 2.2 CT acquisition and image preprocessing

CT scans in HB dataset were performed using Siemens 64-row 128-slice helical CT scan and 40-row 64-slice helical CT scan (SOMATOM Definition AS, tube voltage: 100 kV, tube current: 100 mA, pitch: 1.3, slice thickness: 5.0 mm, field of view (FOV): 430 mm). CT scans in XZ dataset were performed using PHILIPS Brilliance 64-row CT scan (collimator width: 0.75mm, pitch factor: 0.1-2.0, slice thickness: 0.75-2.0mm, scanning parameters 80-140KV, 80-320mAS, A scan matrix: 512 × 512). All CT images were independently reviewed by two radiologists (more than 5-10 years of experience in reading CT images) using LabelImg software with the annotation reference (17). If two Dice coefficient values were all greater than or at least equal to 0.95, they would be averaged as the ground truth of the image. Otherwise, a senior radiologist (more than 20 years of experience in reading CT images) would review and outline the images again to make the final determination. Since the CT imaged were generated by different scanning devices with different resolutions, all data were spatially resampled with the isotropic interval of 1.0 mm × 1.0 mm × 1.0 mm (20).

### 2.3 Development of the baseline segmentation algorithm

**Figure 1:**
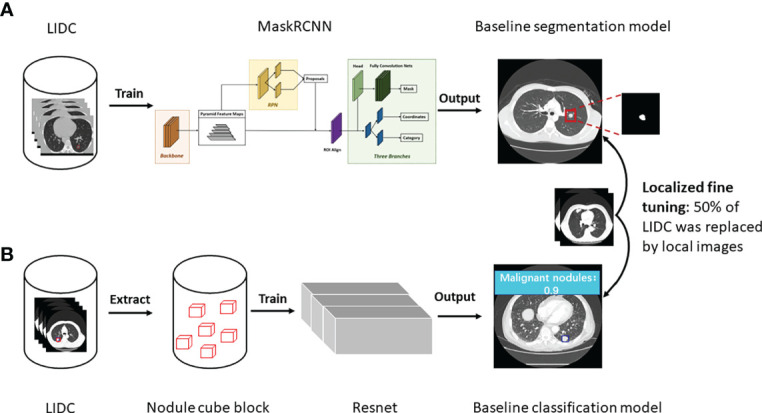
Study workflow. **(A)** The development of baseline and fine tuned segmentation model, and nodules were segmented as the output in the end. **(B)** The development of baseline and fine tuned classification model, and prediction score was given about malignancy or benignity.

As shown in **Figure 1**, 3D MaskRCNN was used to develop a baseline segmentation algorithm to detect and segment nodules on LIDC database. Before inputting the CT images to the network, the input images were transformed to physical millimeter size from pixel size with the spatial location information unchanged. The lung area was first extracted with the remaining part supplemented with pixel of 170 whose neighborhoods are close to one another, where this significantly reduces noise while preserving most image content, and then the images were randomly cropped to the size of [128,128,128] as input training. 3D MaskRCNN is similar to the 2D MaskRCNN which consists of backbone architecture, RPN head and ROI head. The backbone architecture used in the research is resnet50, for which kernels with the size of 3x3x3 were used to convolve the input image, and the feature maps output from it were input into the pooling layer to aggregate contiguous values to one scalar by the mean. The RPN architecture includes a convolutional layer and two following heads which were used to generate every anchor’s shift and the score belonging to foreground, respectively. The ROI align head was involved to pool different proposals to boxes with the shape of 7×7×7, and then a box header and a mask predictor were applied to finetune box position and format the lesion boundary. Specifically, in the research, the image was first input into the backbone and it would output 256 features at a 1/32 ratio of the raw image size, then these features maps were input into the RPN network and 1000 proposals sorted by scores were obtained. Finally, the 1000 proposals were reshaped to 7×7×7 boxes and all the boxes were input into mask head. We ended up selecting the predicted result with a threshold of 0.5. The total training epoch was 200, and ROI Head and Mask Head were added when the epoch was 65 and 80, respectively.

### 2.4 Development of the baseline classification algorithm

Resnet was used to develop the baseline classification algorithm for benign and malignant nodules diagnosis (**Figure 1**). Specifically, first, in the binary classification task of benign and malignant nodules, the center point of the nodule was used as the reference point to extent 64 pixels in the x and y directions, and 32 layers were expanded in the z direction, forming a nodular cube block with the size of [3, 32, 64, 64], which was the input of the algorithm. Then the resnet18-3D was applied to make the calculation of the input of [b, 3, 32, 64, 64], and output [b, 2], where b is the batch size of the algorithm input.

### 2.5 Algorithm fine tuning

For both baseline segmentation algorithm and classification algorithm, 50% of the LIDC training set was replaced by HB and XZ datasets, and then they were trained again to be locally fine tuned, before which the HB and XZ datasets were divided into two parts of sets respectively. For the HB dataset, the one consisting of 172 malignant nodules and 268 benign nodules was used for algorithm fine tuning, and the other set consisting of 365 malignant nodules and 158 benign nodules was used as an independent test. Similarly, for XZ dataset, one set consisting of 172 malignant nodules and 268 benign nodules was used for algorithm fine tuning, and the other set consisting of 215 malignant nodules and 130 benign nodules was used as an independent test. Both baseline algorithms and fine-tuned algorithms were evaluated on HB and XZ independent sets respectively, and their performance were compared in the end(i.e., baseline segmentation algorithm vs. fine-tuned segmentation algorithm; baseline classification algorithm vs. fine-tuned classification algorithm).

### 2.6 Statistical analysis

In the process of evaluating the segmentation algorithm performance, labeled nodules by radiologists are defined as positive findings, and we illustrated segmentation test results by Dice coefficient (DICE), Intersection over Union (IOU), and Average Surface Distance (ASD). For the classification results, the positive findings are malignant nodules and benign nodules are negative, and the receiver operating characteristic (ROC) curve, the value of the area under the ROC curve (AUC), accuracy, sensitivity, specificity and F1 score were used. Statistical analysis was performed using Python 3.8 and SPSS 20. Statistical tests were conducted with p-value< 0.05 as an indicator of statistical significance.

## 3 Results

### 3.1 Clinical characteristics

**Figure 2:**
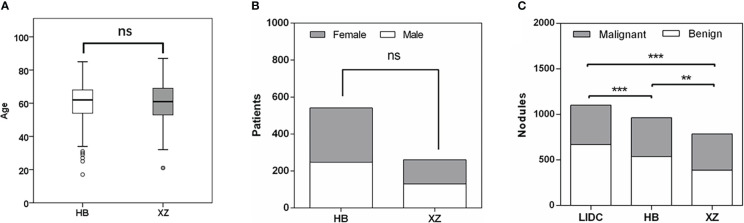
Patients characteristics. **(A)** Age distribution in HB and XZ datasets. **(B)** Gender composition in HB and XZ datasets. **(C)** The composition of malignant nodules and benign nodules in LIDC, HB and XZ datasets. LIDC, lung image database consortium. ns, not significant; **p-value <0.01; ***p-value <0.001.

The main characteristics of patients in the HB and XZ datasets are shown in **Figure 2**. 541 patients from HB dataset were 54.2% males, and the median age was 62 years with an age range of 17-85 years. XZ included 241 patients with 50.2% males (median age of 61 years; age range 21-87 years). There was no significant difference in the patient age (*P* = 0.668) and gender (*P* = 0.292) for both cohorts. However, we observed that the distribution of benign and malignant nodules was statistically significant among LIDC, HB and XZ datasets (*P*<0.001), and the two-two pairwise comparison between any two cohorts also showed significant difference (i.e., LIDC vs. HB: *P*<0.001; LIDC vs. XZ: *P*<0.001; HB vs. HB: *P*=0.007).

### 3.2 Effect of fine tuning on segmentation algorithms

The performance of the baseline and fine tuned segmentation algorithms assessed by the DICE, IOU, and ASD are summarized in **Table 1**. In the internal set of LIDC, the DICE, IOU, ASD of the baseline algorithm were 0.771, 0.642, 0.244, respectively. Then we observed a drop in its performance for external tests, with three metrics being 0.444, 0.348 and 0.304 in HB dataset and 0.486, 0.378 and 0.358 in XZ dataset. Fine tuning enabled the baseline algorithm to perform better on both local datasets, as we observed an increase in the value of DICE and IOU and a decrease in the value of ASD (i.e., 0.593, 0.450 and 0.283 in HB dataset and 0.601, 0.482 and 0.225 in XZ dataset) with corresponding change rate of 33.56%, 29.31% and -6.91% in HB and 23.66%, 27.51% and -37.15% in XZ. Almost all of the change rates are significant except for the -6.91%. Higher values of DICE and IOU, and a lower value of ASD indicate better performance of the segmentation algorithm.

**Table 1:** Performance of baseline and fine tuned segmentation model in public LIDC dataset and two independent collected datasets.

Measure	Performance		
	Datasets		
	LIDC	HB	XZ
Dice coefficient (DICE)			
Baseline algorithm	0.771	0.444	0.486
Fine tuned algorithm	NA	0.593	0.601
Delta in DICE	NA	33.56%	23.66%
*P*	NA	0.021	0.048
Intersection over Union (IOU)			
Baseline algorithm	0.642	0.348	0.378
Fine tuned algorithm	NA	0.450	0.482
Delta in IOU	NA	29.31%	27.51%
*P*	NA	0.029	0.035
Average Surface Distance (ASD)			
Baseline algorithm	0.244	0.304	0.358
Fine tuned algorithm	NA	0.283	0.225
Delta in ASD	NA	-6.91%	-37.15%
*P*	NA	0.067	0.022

LIDC, lung image database consortium; NA, not applicable.

**Figure 3:**
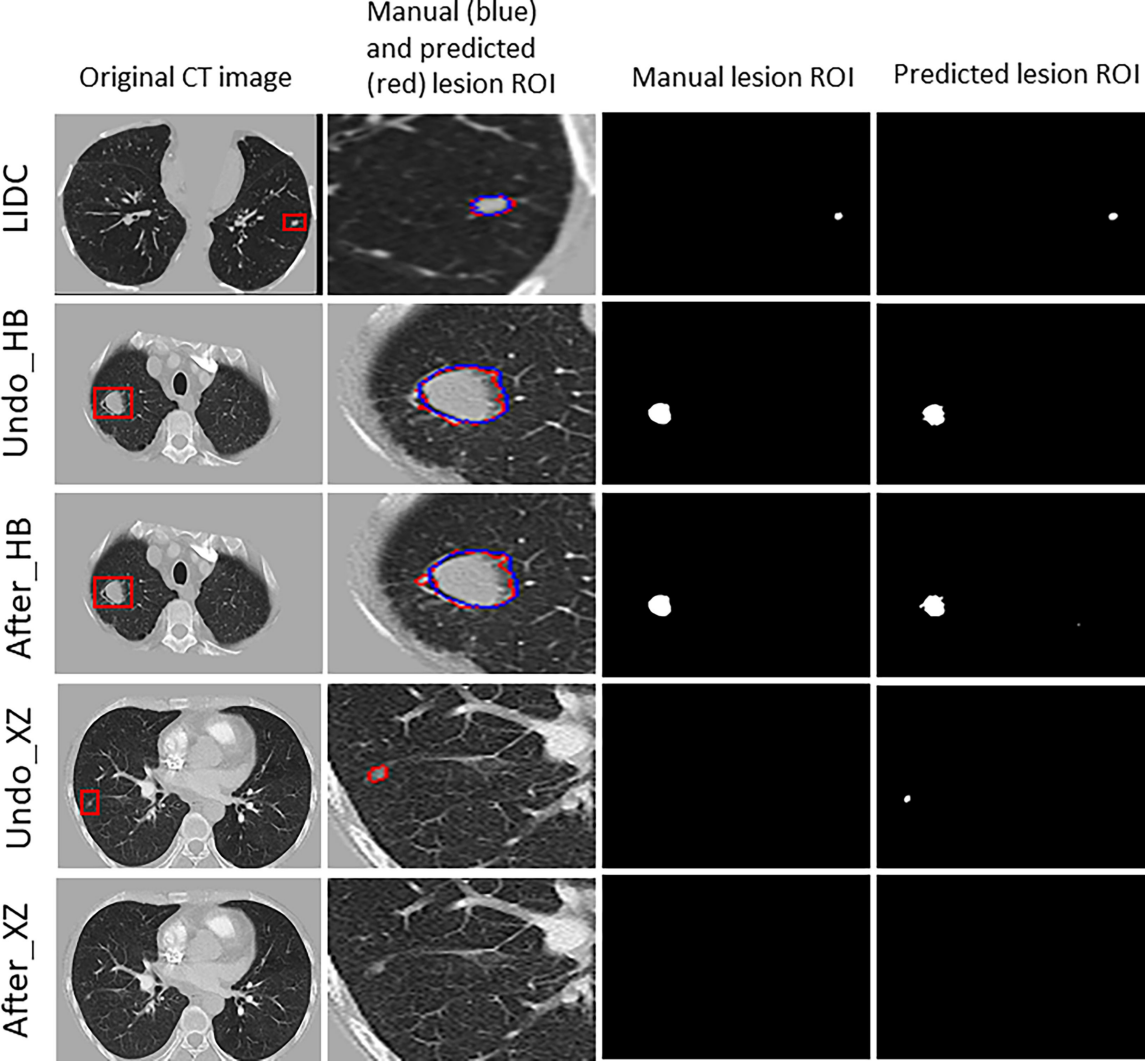
The results for example cases before and after using the localized fine tuning method in pulmonary nodules segmentation, and the manually-labeled ROI (blue contour) was compared to segmentation algorithm predicted ROI (red contour).

**Figure 3** shows examples of segmentation result of the algorithm with and without fine tuning. We observed that the baseline algorithm segmented the lesion region in more details after using the fine tuning method for the HB dataset (i.e., After_HB vs. Undo_HB), which could be reflected by a higher value of ASD that was used to evaluate the algorithms’ edge fitting performance. In addition, it is noteworthy that when the baseline algorithm was applied in XZ dataset, a false positive nodule was detected, but after the algorithm fine tuning the false positive nodule was no longer identified and segmented (i.e., After_XZ vs. Undo_XZ).

### 3.3 Effect of fine tuning on classification algorithms

**Figure 4:**
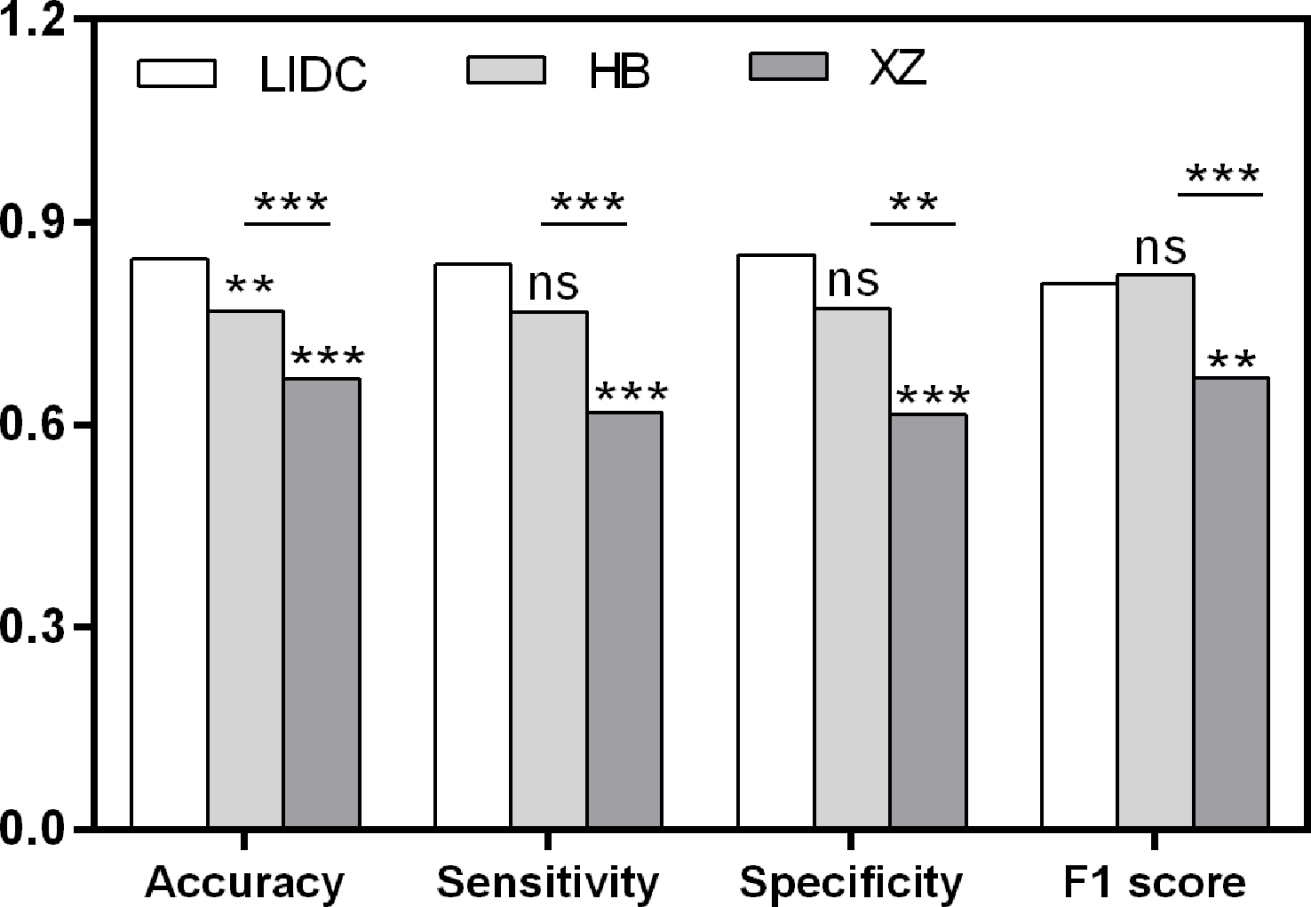
Pairwise performance comparison of the baseline classification model in public LIDC dataset and two independent collected datasets. ns, not significant; **p-value <0.01; ***p-value <0.001. LIDC, lung image database consortium.

**Figure 5:**
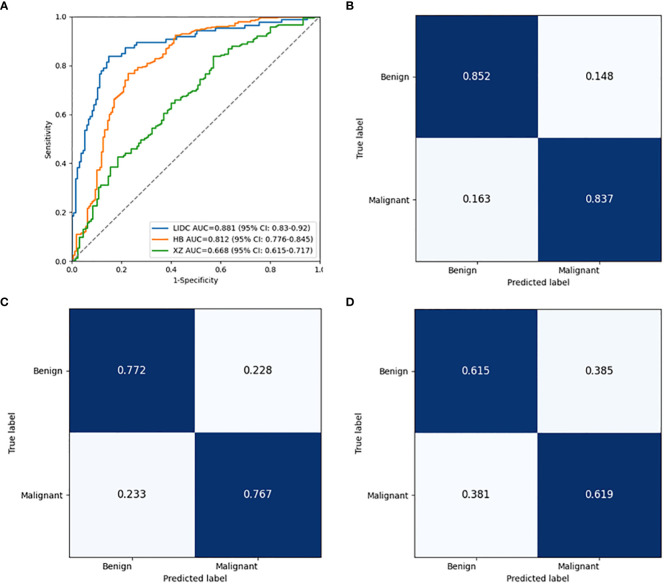
Performance comparison of the baseline classification model in public LIDC dataset and two independent collected datasets. **(A)** The ROC curves in LIDC, HB and XZ datasets. **(B)** Normalized confusion matrix in LIDC dataset. **(C)** Normalized confusion matrix in HB dataset. **(D)** Normalized confusion matrix in XZ dataset. ROC, receiver operating characteristic; LIDC, lung image database consortium.

As shown in **Table 2**, the baseline classification algorithm achieved an AUC of 0.881, and the accuracy was 0.846 in the internal testing. When it was applied in two local datasets, the AUC decreased to 0.812 and 0.668, and the accuracy decreased to 0.769 and 0.617 in HB and XZ datasets, respectively. Other metrics of sensitivity, specificity and F1 score also experienced a decreasing tendency in both HB and XZ datasets. However, they exhibited varying degrees of decrease (**Figures 4**, **5**), which is consistent with prior research revealing that the proposed algorithm would display high variability in performance across external datasets (18).

**Table 2:** Performance of baseline classification model in both public dataset and independent collected datasets.

Measure	Performance (95% CI)		
	Datasets		
	LIDC	HB	XZ
AUC	0.881 (0.830-0.920)	0.812 (0.776-0.845)	0.668 (0.615-0.717)
Accuracy	0.846 (0.792-0.888)	0.769 (0.731-0.803)	0.617 (0.565-0.667)
Sensitivity	0.837 (0.744-0.902)	0.767 (0.721-0.808)	0.619 (0.552-0.681)
Specificity	0.852 (0.782-0.903)	0.772 (0.700-0.831)	0.615 (0.530-0.695)
F1 score	0.809 (0.789-0.828)	0.822 (0.803-0.840)	0.668 (0.621-0.713)

AUC, area under the ROC curve.

**Figure 6:**
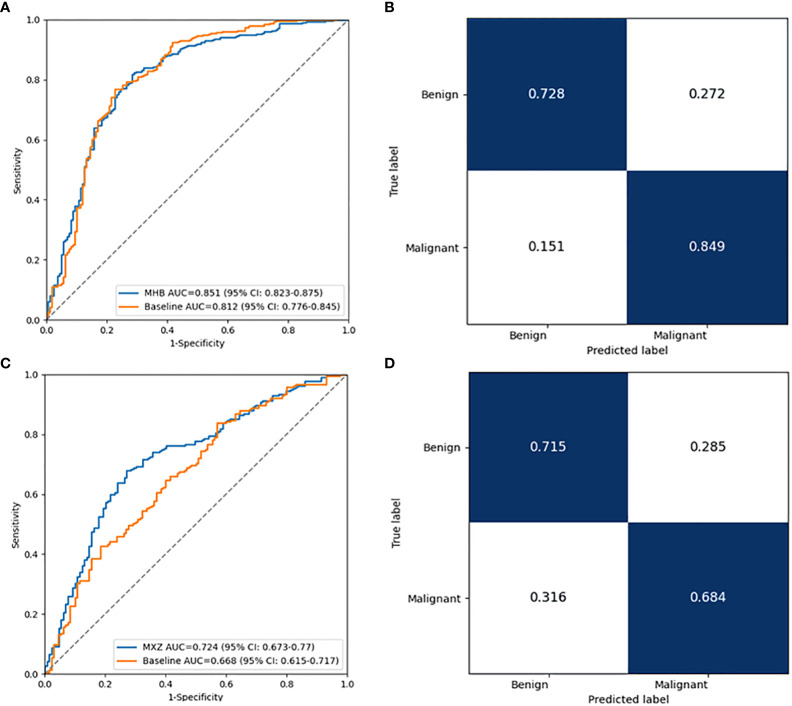
Performance comparison between the baseline classification model and fine tuned classification model in two independent collected datasets. **(A)** The ROC curves of the two models in HB dataset. **(B)** Normalized confusion matrix of the fine tuned model in HB dataset. **(C)** The ROC curves of the two models in XZ dataset. **(D)** Normalized confusion matrix of the fine tuned model in XZ dataset. ROC, receiver operating characteristic.

To explore the effect of fine tuning on classification algorithm, the comparison of the validation results between baseline algorithms and fine tuned algorithms, namely MHB and MXZ, was conducted (**Table 3**). The classification performance of both MHB and MXZ was improved after the fine tuning (**Figure 6**). Specifically, comparing with the baseline validation results, the MHB had higher AUC (0.851 vs. 0.812), accuracy (0.813 vs. 0.769), sensitivity (0.849 vs. 0.767) and F1 score (0.852 vs. 0.822), and their change rate were 4.8%, 5.7%, 10.7% and 3.6%. Though the specificity was slightly decreased by 5.4%, there was no significant difference (0.730 vs. 0.772, *P*=0.363). For MXZ validation results, all the evaluating metrics were increased, including AUC (0.724 vs. 0.668), accuracy (0.696 vs. 0.617), sensitivity (0.684 vs. 0.619), specificity (0.713 vs. 0.615) and F1score (0.737 vs. 0.668), and their change rate were 8.4%, 12.8%, 10.5%, 15.9% and 10.3%.

**Table 3:** Comparison of the baseline classification model and its fine tuned models.

	AUC (95% CI)	Accuracy (95% CI)	Sensitivity (95% CI)	Specificity (95% CI)	F1 score (95% CI)
Baseline	0.812 (0.776-0.845)	0.769 (0.731-0.803)	0.767 (0.721-0.808)	0.772 (0.700-0.831)	0.822 (0.803-0.840)
MHB	0.851 (0.823-0.875)	0.813 (0.777-0.844)	0.849 (0.809-0.883)	0.730 (0.654-0.791)	0.852 (0.846-0.879)
Rate of change	4.8%	5.7%	10.7%	-5.4%	3.6%
*P*	0.011	0.080	0.005	0.363	0.030
Baseline	0.668 (0.615-0.717)	0.617 (0.565-0.667)	0.619 (0.552-0.681)	0.615 (0.530-0.695)	0.668 (0.621-0.713)
MXZ	0.724 (0.673-0.770)	0.696 (0.645-0.742)	0.684 (0.619-0.742)	0.713 (0.632-0.786)	0.737 (0.714-0.759)
Rate of change	8.4%	12.8%	10.5%	15.9%	10.3%
*P*	0.096	0.030	0.157	0.088	0.034

AUC, area under the curve.

## 4 Discussion

In this study, we developed a baseline segmentation algorithm and a baseline classification algorithm with public dataset of LIDC to segment nodules and classify them as being benign or malignant, and then conducted fine tuning for both of them to compare their performance with that of their baseline ones. The results showed that both segmentation and classification process benefit from fine tuning and end up obtaining higher performance for the site-specific use.

Generally, the development of a computer-aided diagnosis (CAD) scheme consists of the following steps: image preprocessing, ROI segmentation, feature extraction, and finally classification. DL models have been shown to significantly contribute to medical image analysis for the processes of segmentation and classification (21), and many methods have been proposed on optimizing the segmentation and classification algorithm independently (22). Technically, segmentation is used to detect and localize the ROI from the background within the medical image, followed by the segment-based classification task to classify the ROI to a certain class, and the DL model performance may largely rely on the reliable ROI segmentation and good classifier (23). In the current study, we first proposed baseline DL algorithms of segmentation and classification, and compared the performance before and after fine tuning on imaging to explore to what extent the fine tuning can help improve the segmentation and classification process independently.

Algorithms developed on public datasets may not be implied directly on other populations, and rigorous external validation is essential to objectively assess the performance of a detection algorithm (24). In the study, we developed a segmentation and a classification algorithm using public dataset of LIDC, and unlike most of the work with adopted and balanced validation dataset, we applied two external datasets which are inherently different from each other with a significant difference in the distribution of benign and malignant nodules. Thus, the algorithm performance was evaluated in the real-word screening setting, providing objective evidence for the usefulness of the algorithm. It is common to conduct a pilot phase to optimize a triaging threshold of CAD system for external test. However, the threshold choice is balanced between maximal case finding and lower false positive cases without model improvement (25, 26). Therefore, in the study, even with the optimal threshold we observed a decreased performance in the two external tests for both baseline segmentation and classification algorithm (**Table 2**). The results showed that the algorithm trained by public dataset needs further adjustments for site-specific use, which is consistent with reported research (27, 28).

In previous studies, the deep learning models used for lung nodules segmentation on LIDC dataset obtained the DICE values of over 0.6 (29), and the existed classification algorithm had AUC values of over 0.8 for benign and malignant nodules classification (5, 30, 31), which is similar to our baseline segmentation algorithm and baseline classification algorithm. However, the DICE value decreased when the baseline segmentation algorithm was applied on HB and XZ, and the performance drop could also be detected in the external tests for the baseline classification algorithm. This may result from the significant appearance variances caused by the population and setting differences (32–34). It has been reported that involving multi-center datasets to develop algorithm is effective to keep the algorithm robust to maintain its accuracy across datasets (10, 11). However, it is unclear how many datasets should be exactly included to create a robust detection algorithm to obtain comparable performances of the internal test, especially when those external datasets are significantly different from internal datasets. Furthermore, AlBadawy et al. reported that using multiple institutions for training does not necessarily remove the dataset shift limitation (32). Model tuning with additional data from specific settings may be an effective way to reduce site-specific biases (11) but few studies revealed its impact on segmentation and classification process alone. In the current study, the baseline models trained by public data set were fine tuned with site-specific images and we observed both segmentation and classification algorithm benefit from the fine tuning, which showed that localized fine tuning would be a potential and well-operated way to develop an automated diagnostic tool to screen lung cancer as both the segmentation process and classification process could get optimized (35). It should be noted that the baseline segmentation algorithm was fine tuned to have as high of a sensitivity as possible for localizing and segmenting the nodules, allowing for false positive reduction, which might be due to that homogeneous features of the local dataset were involved for the learning process.

There are some limitations to this study. First, although both segmentation process and classification process were found improved with the fine tuning, it only focused on lung nodules. For the next step of our study, we aim to expand to other lung abnormality/disease to comprehensively validate the effectiveness of the fine tuning method. Second, the current study was a retrospective study where both LIDC and two collected datasets were available at the time of study, therefore, a prospective evaluation is needed to further validate the proposed method.

## Conclusion

Our work is among the first that conducted the localized fine tuning for DL algorithm on imaging to explore its impact on the segmentation and classification process respectively. Results showed that both segmentation and classification algorithm outperformed their baseline model, which might enable a baseline algorithm be generalized for site-specific use and promote the future in-depth research towards its clinical application.

## Data availability statement

The raw data supporting the conclusions of this article will be made available from corresponding author on reasonable request.

## Ethics statement

The studies involving human participants were reviewed and approved by the Institutional Review Board (IRB) of the Affiliated Hospital of Hebei University. The informed consent from human participants was waived because this is a retrospective study, and the waiver was indicated in the IRB approval document.

## Author contributions

Study design and conception were proposed by JC, LG, LZ and XY. Paper writing was done by JC, LG and LZ. CT scans were collected by JC and LG. AI model training, testing and visualizing were done by LX, LQ and YMFL. All authors interpreted the results and revised the manuscript. All authors read and approved the final manuscript. All authors contributed to the article and approved the submitted version.
